# Supplementary material for: Implementation of mercury biomonitoring in German adults using dried blood spot sampling in combination with direct mercury analysis
Source: Environ Monit Assess. 2021 Jul 10;193(8):488. doi: 10.1007/s10661-021-09254-0 (PMC8272700; doi:10.1007/s10661-021-09254-0)

**Supporting Information**

**Calculation of the limits of detection (LOD) and quantitation (LOQ)**

The limits of detection (LOD) and quantitation (LOQ) have been calculated based on the analysis of blank values to determine the amount of Hg that is significantly different from the blank value. Therefore, 20 blank samples for venous blood (just sample boats) and DBS samples (sample boat and blank 0.5 inch DBS punch) were analyzed (**Table S1**). The mean Hg background and the variance of the blank signal was used for LOD/LOQ calculation. In detail, the meand and the standard deviation (SD) were translated into an absolute amount of Hg. For the LOD, 3 times the SD was added to the mean background, for LOQ 10 times the SD was added to the mean background. Finally, absolute Hg amounts were converted into concentrations by taking the analyzed sample volume (venous blood: 100 µl, DBS: 60 µl) into account (**Table S1**).

During the stability tested and the field study, many more blank DBS spots were analyzed. **Figure S2** summarizes the data.

Table S1: Blank signals for venous blood (just sample boats) and DBS (sample boat and blank DBS), mean and standard deviation of blank signals and translation into LOD/LOQ. Absolute Hg levels were calculated by using the external calibration. Hg concentrations were calculated with respect to the sample volume (venous blood: 100 µl, DBS: 60 µl).

|  | **blank signals** | |
| --- | --- | --- |
|  | **venous blood** | **DBS** |
|  | 0.0002 | 0,0006 |
|  | 0.0003 | 0,0004 |
|  | 0.0002 | 0,0006 |
|  | 0.0002 | 0,0004 |
|  | 0.0002 | 0,0008 |
|  | 0.0002 | 0,0004 |
|  | 0.0002 | 0,0006 |
|  | 0.0002 | 0,0006 |
|  | 0.0003 | 0,0008 |
|  | 0.0002 | 0,0005 |
|  | 0.0002 | 0,0006 |
|  | 0.0002 | 0,0005 |
|  | 0.0002 | 0,0004 |
|  | 0.0002 | 0,0005 |
|  | 0.0002 | 0,0007 |
|  | 0.0002 | 0,0007 |
|  | 0.0002 | 0,0005 |
|  | 0.0003 | 0,0006 |
|  | 0.0002 | 0,0011 |
| mean (signal) | 0.000215 | 0.000605 |
| mean (ng) | 0.001540 | 0.004333 |
| RSD (signal) | 0.000037 | 0.000176 |
| RSD (ng) | 0.000262 | 0.001262 |
| LOD (µg/l) | 0.02 | 0.14 |
| LOQ (µg/l) | 0.04 | 0.28 |

Table S2: Hg levels in venous blood samples stratified by gender, age, fish consumption, and dental amalgam. All Hg levels are given in µg/l.

|  |  | **n** | **GM** | **Median** | **Min** | **Max** |
| --- | --- | --- | --- | --- | --- | --- |
| **Gender** | **Men** | 24 | 0.65 | 0.69 | 0.13 | 3.55 |
|  | **Women** | 26 | 0.65 | 1.07 | < LOD | 4.35 |
| **Age** | **18 – 30 years** | 32 | 0.55 | 0.75 | < LOD | 4.35 |
|  | **30 – 45 years** | 8 | 0.72 | 0.85 | 0.18 | 2.26 |
|  | **45 – 60 years** | 8 | 1.01 | 1.12 | 0.24 | 2.49 |
|  | **>60 years** | 2 | 0.97 | 0.96 | 0.89 | 1.06 |
| **Fish consumption** | **Never** | 7 | 0.10 | 0.18 | < LOD | 0.30 |
|  | **Up to once per month** | 12 | 0.43 | 0.47 | 0.13 | 1.85 |
|  | **2-4 times per month** | 26 | 1.06 | 1.13 | 0.24 | 4.35 |
|  | **>1 time per week** | 5 | 1.97 | 2.32 | 0.89 | 3.55 |
| **Dental amalgam fillings** | **yes** | 10 | 0.69 | 0.83 | 0.18 | 2.43 |
|  | **no** | 39 | 0.64 | 0.85 | < LOD | 4.35 |
|  | **missing** | 1 |  | | | |

GM: geometric mean

Tables S3: Individual mean Hg levels [µg/l] in venous blood and the corresponding DBS samples (n =50, samples were analyzed in triplicate where available).

| **venous blood** | **corresponding DBS** | **venous blood** | **corresponding DBS** |
| --- | --- | --- | --- |
|  |  |  |  |
| < LOD | < LOQ | 0.88 | 1.09 |
| < LOQ | < LOQ | 0.89 | 0.90 |
| 0.13 | < LOQ | 0.94 | 0.83 |
| 0.15 | 0.31 | 1.06 | 1.50 |
| 0.15 | < LOQ | 1.07 | 0.69 |
| 0.18 | < LOQ | 1.08 | 0.66 |
| 0.23 | 0.31 | 1.10 | 0.68 |
| 0.24 | 0.36 | 1.16 | 1.19 |
| 0.24 | 0.38 | 1.17 | 1.10 |
| 0.27 | < LOQ | 1.18 | 1.08 |
| 0.29 | 0.36 | 1.25 | 1.03 |
| 0.30 | 0.27 | 1.28 | 1.37 |
| 0.38 | 0.51 | 1.35 | 0.89 |
| 0.41 | 0.43 | 1.39 | 1.20 |
| 0.42 | 0.47 | 1.42 | 1.38 |
| 0.47 | 0.31 | 1.68 | 1.46 |
| 0.47 | 0.54 | 1.85 | 1.58 |
| 0.48 | 0.57 | 1.85 | 1.24 |
| 0.52 | 0.64 | 1.96 | 1.81 |
| 0.55 | 0.35 | 2.26 | 1.85 |
| 0.63 | 0.48 | 2.32 | 1.96 |
| 0.75 | 0.82 | 2.43 | 2.53 |
| 0.78 | 0.55 | 2.49 | 2.01 |
| 0.79 | 0.76 | 3.55 | 3.12 |
| 0.85 | 1.20 | 4.35 | 3.18 |

LOD: Limit of detection, LOQ: Limit of quantitation

Figure S1: Storage of DBS samples in plastic bags (right) and glass tubes (left).


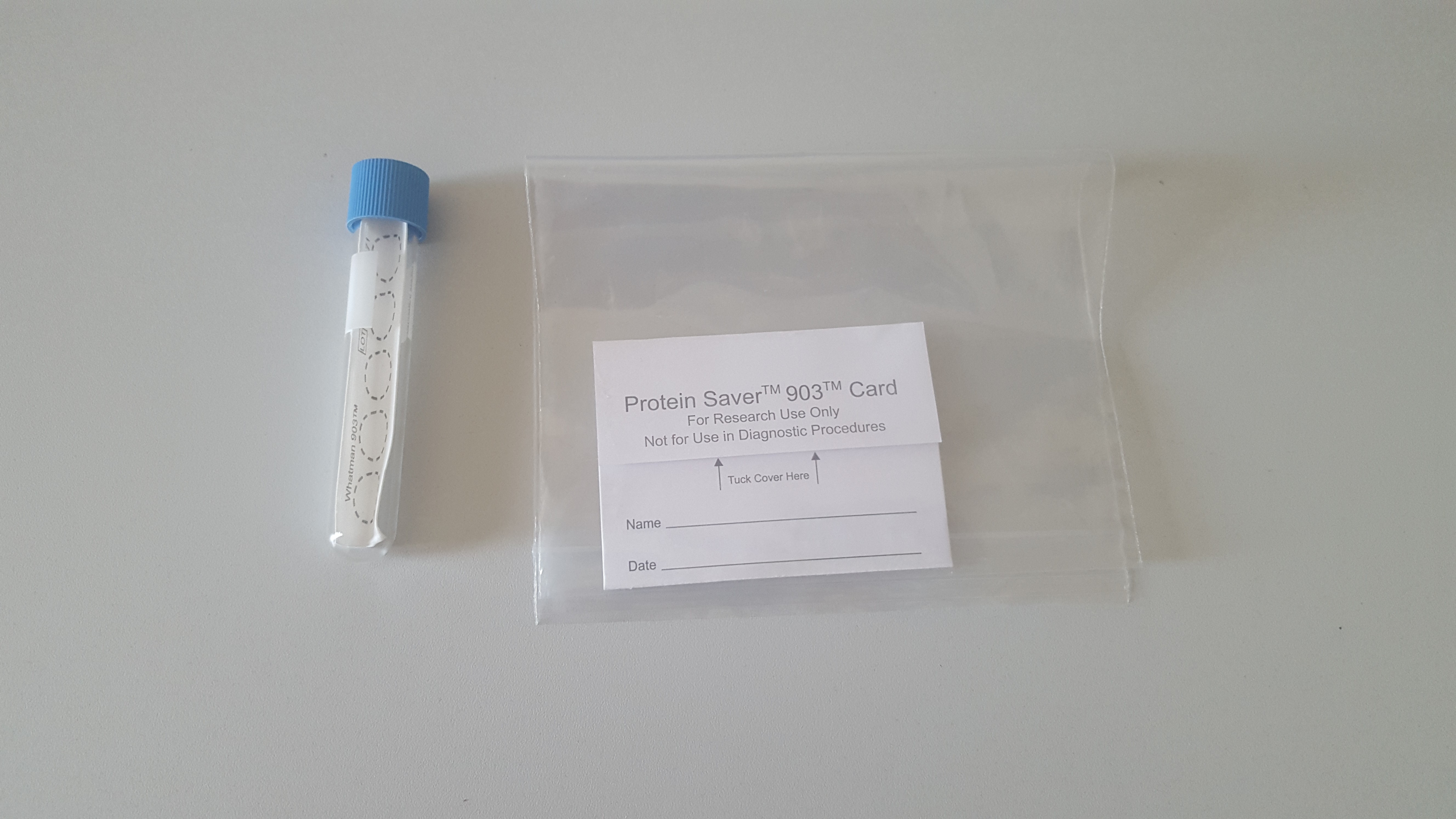


Figure S2: Mean signal heights and relative standard deviations of DBS blanks during sampling phase


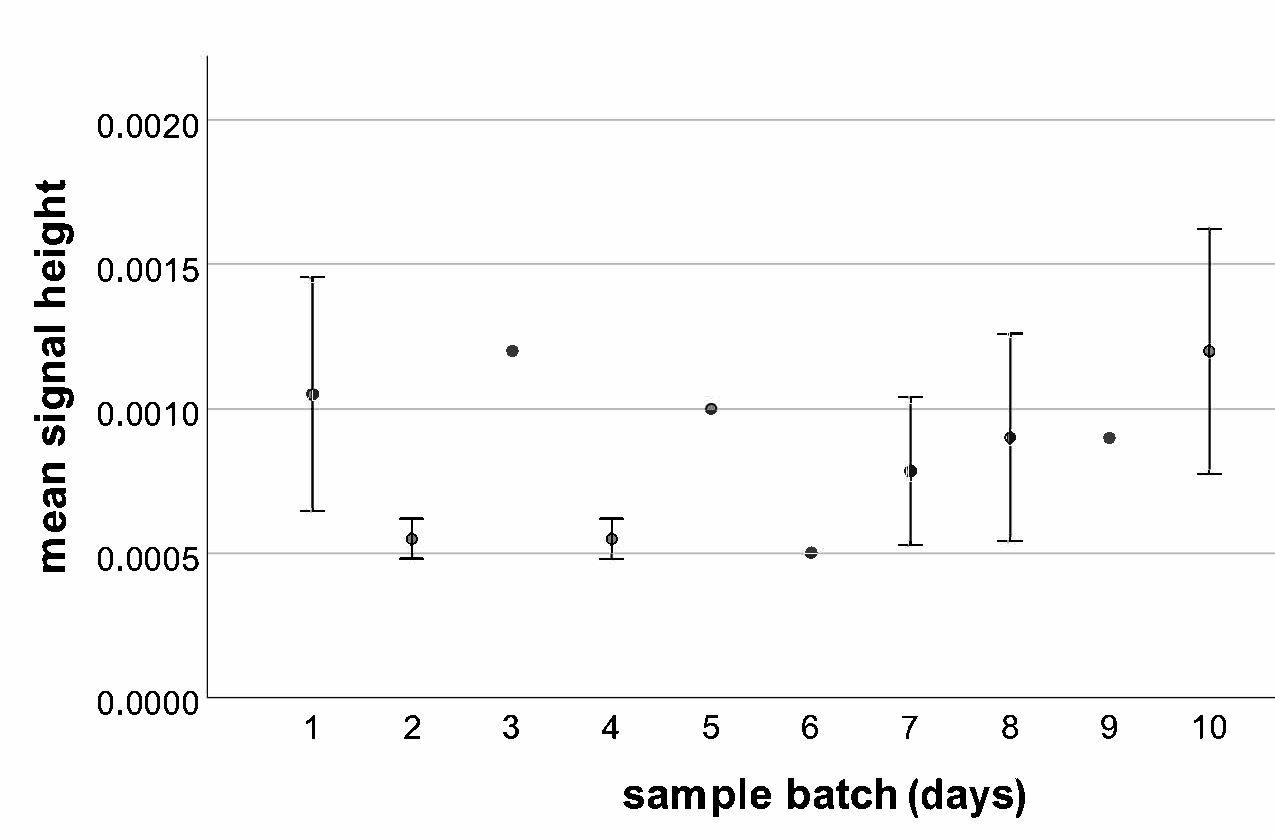


Figure S3: Effect of storage conditions (temperature, time) on the Hg recovery in DBS cards stored in uncleaned glass tubes. The bar graph show the mean recoveries from six individual DBS spots and the error bars (standard deviation). Results from the experiment -20 °C/2 Weeks are missing due to a contamination during sample handling.


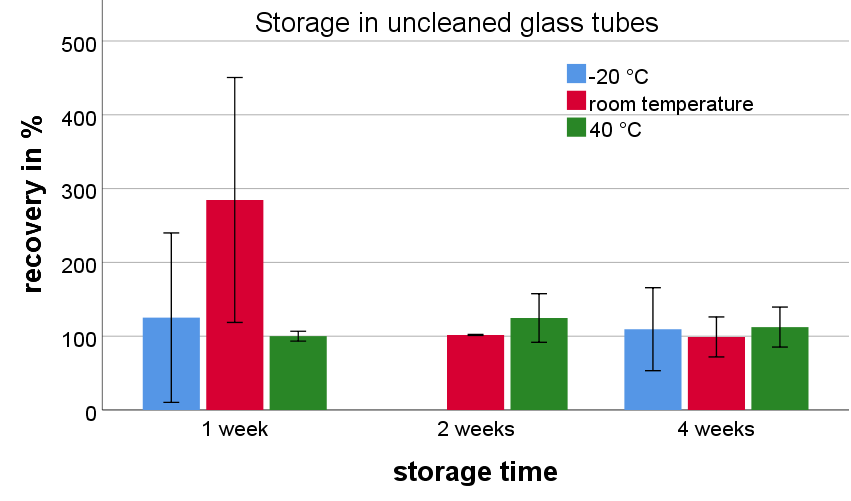


Figure S4: Effect of storage time on the Hg recovery in DBS cards stored in plastic bags at 40 °C. The bar graph show the mean recoveries from three individual DBS spots and the standard deviation. DBS cards were prepared using two certified reference materials for blood (ClinChek^®^).


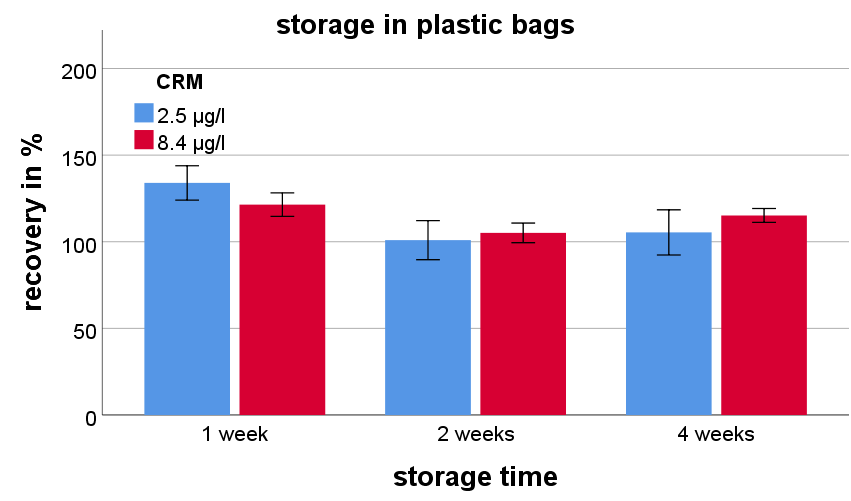

Supplement: Supplementary file 1 — Supplementary file1 (DOCX 2614 KB) [file 10661_2021_9254_MOESM1_ESM.docx]
